# Delta-like 1 regulates Bergmann glial monolayer formation during cerebellar development

**DOI:** 10.1186/1756-6606-6-25

**Published:** 2013-05-21

**Authors:** Yuichi Hiraoka, Okiru Komine, Mai Nagaoka, Ning Bai, Katsuto Hozumi, Kohichi Tanaka

**Affiliations:** 1 Laboratory of Molecular Neuroscience, Medical Research Institute, Tokyo Medical and Dental University, 1-5-45 Yushima, Bunkyo-ku, Tokyo 113-8510, Japan; 2 Department of Immunology, Tokai University School of Medicine, 143 Shimokasuya, Isehara-shi, Kanagawa 259-1193, Japan; 3 The Center for Brain Integration Research, Tokyo Medical and Dental University, Tokyo, Japan; 4 JST, CREST, Saitama, Japan

**Keywords:** Bergmann glia, Notch signaling, Delta like 1, Conditional knockout mouse, Monolayer formation

## Abstract

**Background:**

Bergmann glia (BG) are unipolar cerebellar astrocytes. The somata of mature BG reside in the Purkinje cell layer and extend radially arranged processes to the pial surface. BG have multiple branched processes, which enwrap the synapses of Purkinje cell dendrites. They migrate from the ventricular zone and align next to the Purkinje cell layer during development. Previously, we reported that *Notch1*, *Notch2*, and *RBPj* genes in the BG play crucial roles in the monolayer formation and morphogenesis of BG. However, it remains to be determined which ligand activates Nocth1 and Notch 2 on BG. Delta-like 1 (Dll1) is a major ligand of Notch receptors that is expressed in the developing cerebellum.

**Results:**

In this study, we used human glial fibrillary acidic protein (hGFAP) promoter-driven Cre-mediated recombination to delete Dll1 in BG. Dll1-conditional mutant mice showed disorganization of Bergmann fibers, ectopic localization of BG in the molecular layer and a reduction in the number of BG.

**Conclusion:**

These results suggest that Dll1 is required for the formation of the BG layer and its morphological maturation, apparently through a Notch1/2-RBPj dependent signaling pathway.

## Background

Bergmann glia (BG) are unipolar cerebellar astroglial cells that have their soma in the Purkinje cell layer and extend radially arranged processes to the pial surface [1]. Purkinje cells constitute the sole output of all motor coordination in the cerebellar cortex. Mature BG processes ensheath Purkinje cell somata, dendrites, and both excitatory and inhibitory synapses. BG express GABA and glutamate transporters, which in turn are involved in their clearance from the synapses. A recent study demonstrated that photoactivation of BG can modulate the neuronal activity of Purkinje cells by releasing of glutamate [2]. Thus, BG play important roles in the adult cerebellar cortex. During development, BG are among the earliest cells to develop in the cerebellum, and their radial processes assist in the migration of Purkinje and granule cells during the construction of the cerebellum [3,4]. BG arise from neuroepithelial cells in the fourth ventricle and migrate from the ventricular zone through the mantle zone in synchrony with the migration of Purkinje cells, where they form a monolayer structure in the Purkinje cell layer from embryonic day (E) 14 to postnatal day (P) 7 in the mouse cerebellum [5]. However, the molecular mechanisms involved in the development and differentiation of BG remain unclear.

The Notch signaling pathway is among the most evolutionally conserved signaling pathways in various animals. The Notch signaling is characterized as mediating cell–cell signaling between adjacent cells. In the central nervous system (CNS), Notch signaling regulates neural progenitor maintenance and differentiation. In the traditional view, Notch receptor activation inhibits neurogenesis in order to maintain neural stem and/or progenitor cell character and that Notch may play an instructive role in promoting glial development [6,7]. We have previously reported that ablation of Notch1/2 or RBPj in BG results in poor radial fiber extension and defective positioning of BG in the adult cerebellum [8]. This implies that Notch signaling activation is crucial for BG monolayer formation and its morphological development, however, precisely how the Notch pathway is activated remains unclear. During early postnatal development, Dll1 and Jagged-1 are predominant Notch ligands expressed in the cerebellum [9]. It has been reported that conditional knockout of Jagged1 from the neuroepithelial cells of the midbrain–hindbrain boundary causes a reduction in the number and processes of BG, however, the mutant mice did not display abnormal localization of BG in the molecular layer [10]. Therefore, Dll1 is a candidate gene for the ligand that plays an indispensable role in BG monolayer formation. Dll1 mRNAs were present in the molecular and internal granular layer during early postnatal stages, suggesting that Dll1 was expressed in interneurons of the molecular layer, Purkinje cells, BG and granule cells. To address this question, we used a hGFAP-Cre transgenic mouse line and employed the Cre-loxP approach to inactivate Dll1 in BG. Loss of Dll1 resulted in disorganization of Bergmann fibers, irregularities in the BG lining and aberrant localization of BG in the molecular layer, thus displaying phenotypic similarities with Notch1/2 or RBPj conditional knockout mice.

## Results

### Conditional ablation of Dll1 leads to anatomical abnormalities in the adult cerebellum

**Figure 1:**
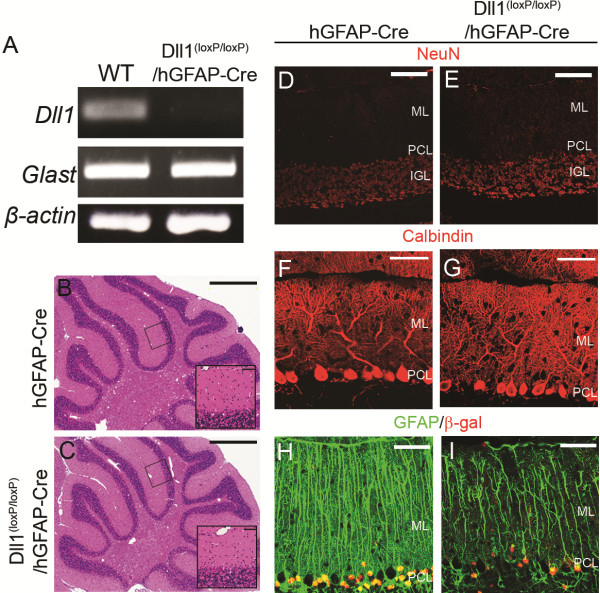
**Phenotypic analysis of adult Dll1**^**(loxP/loxP)**^**/hGFAP-Cre mice.** (**A**) RT-PCR analysis shows the Cre-mediated deletion of Dll1 in cultured astrocytes from Dll1^(loxP/loxP)^/hGFAP-Cre mice cerebella. (**B, C**) Hematoxylin and eosin stained sagittal sections from hGFAP-Cre (**B**) and Dll1^(loxP/loxP)^/hGFAP-Cre (**C**) mice. Boxed areas are shown as higher magnifications in insets in (**B**) and (**C**). (**D**, **E**) Granule cells labeled by an anti-NeuN antibody (red) in hGFAP-Cre (**D**) and Dll1^(loxP/loxP)^/hGFAP-Cre (**E**) mice. (**F**, **G**) Purkinje cells labeled by an anti-calbindin antibody (red) in hGFAP-Cre (**F**) and Dll1^(loxP/loxP)^/hGFAP-Cre (**G**) mice. (**H**, **I**) BG labeled by anti-GFAP (green) and anti-β-galactosidase (red) antibodies in hGFAP-Cre (**H**) and Dll1^(loxP/loxP)^/hGFAP-Cre (**I**) mice. Scale Bars: 1 mm (**B**, **C**), 50 μm (Insets in **B** and **C**), 100 μm (**D**, **E**), 50 μm (**F**-**I**). IGL, internal granular layer; ML, molecular layer; PCL, Purkinje cell layer.

To study Dll1 function during cerebellar development, we inactivated Dll1 by using the Cre-loxP system with hGFAP-Cre mice, whereby the human GFAP promoter directs the expression of Cre recombinase. To demonstrate the deletion of Dll1 in astrocytes, we prepared primary astrocytic cultures. RT-PCR analysis revealed the Cre-mediated deletion of Dll1 in cultured astrocytes that were derived from the cerebellum of Dll1^(loxP/loxP)^/hGFAP-Cre mice (Figure 1A). The Dll1^(loxP/loxP)^/hGFAP–Cre mice survived to adulthood and displayed no obvious abnormalities compared with controls. To determine the effect of Dll1 deletion on the cerebellum, we examined its gross anatomical organization in adult Dll1^(loxP/loxP)^/hGFAP–Cre mice, however no significant difference was observed between the cerebellum of Dll1^(loxP/loxP)^/hGFAP-Cre and control mice (Figure 1B-G). To assess the effect of Dll1 loss on BG, we employed immunohistochemistry to analyze GFAP expression on BG processes in adult mice. In the controls, the glial processes extended to the surface of the cerebellum where they formed end feet (Figure 1H). In contrast, the number of glial processes in Dll1^(loxP/loxP)^/hGFAP–Cre mice was significantly decreased and failed to extend to the pial surface of the cerebellum (Figure 1I). On the other hand, we did not observe any obvious defects of other cell types in these mice. The hematoxylin and eosin stains revealed that the number of cells in the molecular layer (stellate and basket cells) was normal in the mutant mice (Figure 1B, C). Immunostaining of a granule cell marker, neuronal nuclei protein (NeuN), showed that the granule cell layer of the mutants was of normal thickness and cell density in comparison to that of the control mice (Figure 1D, E). Calbindin immunohistochemical labeling of Purkinje cells showed that the number and morphology of Purkinje cells did not differ between the control and the mutant mice (Figure 1F, G). Taken together, these results suggest that the BG development is impaired in Dll1^(loxP/loxP)^/hGFAP–Cre mice, while other cell types develop normally.

### Adult Dll1^(loxP/loxP)^/hGFAP–Cre mice show defects in BG

**Figure 2:**
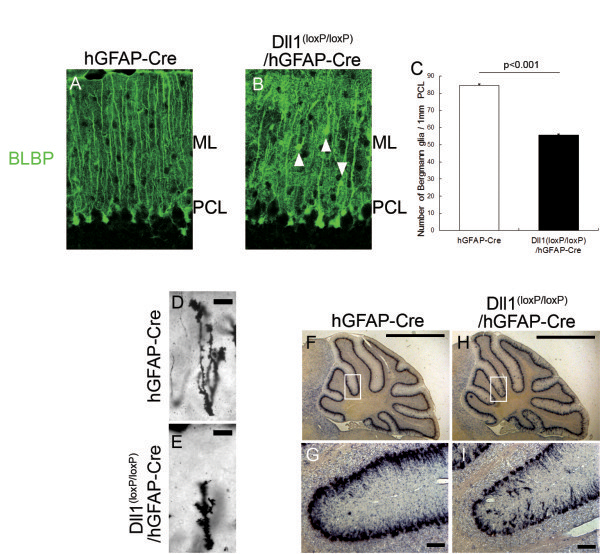
**Abnormal BG in adult Dll1**^**(loxP/loxP)**^**/hGFAP-Cre mice.** (**A, B**) Immunohistochemistry for BLBP (green) in hGFAP-Cre (**A**) and Dll1^(loxP/loxP)^/hGFAP-Cre (**B**) mice. The white arrowheads in (**B**) show ectopic BG. (**C**) Quantification of the number of BLBP-positive cell bodies. (**D**, **E**) Morphology of BG by Golgi-staining in hGFAP-Cre (**D**) and Dll1^(loxP/loxP)^/hGFAP-Cre (**E**) mice. (**F**-**I**) in situ hybridization for GLAST mRNA in hGFAP-Cre (**F**, **G**) and Dll1^(loxP/loxP)^/hGFAP-Cre (**H**, **I**) mice. The red arrowheads in (**G**, **I**) show ectopic BG. Scale bars: 50 μm (**A**, **B**), 10 μm (**D**,**E**), 1 μm (**F**,**H**), 50 μm (**G**, **I**). ML, molecular layer; PCL, Purkinje cell layer.

To determine the effect of the knocking out Dll1 in BG, we first examined the number of BG by immunohistostaining for brain lipid-binding protein (BLBP) in the adult Dll1^(loxP/loxP)^/hGFAP–Cre mice (Figure 2A, B). We found a significant reduction in the number of BG in the Dll1^(loxP/loxP)^/hGFAP–Cre mice compared to their control littermates (Figure 2C). Next, we examined the morphology of BG. In the control cerebellum, BG fibers were well organized and had an exclusively radial orientation, in contrast, BG fibers in the Dll1^(loxP/loxP)^/hGFAP–Cre mice were truncated and failed to extend to the pial surface (Figure 2A, B). Golgi staining showed that the radial processes looked thicker and disorganized, and the complexity of their fine lateral processes was visibly reduced in Dll1^(loxP/loxP)^/hGFAP–Cre mice (Figure 2D, E). Furthermore, the monolayer alignment of BG was disrupted and a considerable number of BG were frequently misplaced in the molecular layer of Dll1^(loxP/loxP)^/hGFAP-Cre mice, as revealed by immunohistochemistry of BLBP (Figure 2B) and in situ hybridization of *Glast* (Figure 2F-I). These results indicate that the deletion of Dll1 causes a reduction in the number of BG, as well as affects their arrangement and morphology in the cerebellum.

### Lack of Dll1 produces alterations in the arrangement of BG in the purkinje cell layer during early postnatal stages

**Figure 3:**
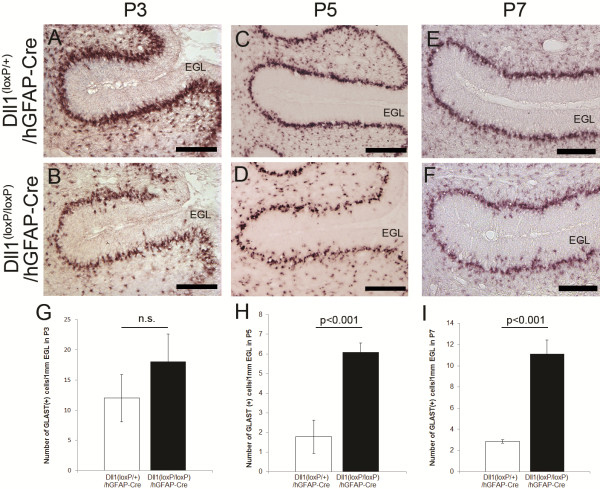
**Impaired monolayer formation of BG in Dll1(loxP/loxP)/hGFAP-Cre mice during cerebellar development.** (**A-F**) In situ hybridization for GLAST at P3 (**A**, **B**), P5 (**C**, **D**), and P7 (**E**, **F**) in Dll1^(loxP/+)^/hGFAP-Cre (**A**, **C**, **E**) and Dll1^(loxP/loxP)^/hGFAP-Cre (**B**, **D**, **F**) mice. (**G-I**) Quantification of the number of GLAST-positive cells in EGL at P3 (**G**), P5 (**H**) and P7 (**I**). n.s., not significant. Scale bars: 100 μm (**A-F**). EGL, external granular layer.

**Figure 4:**
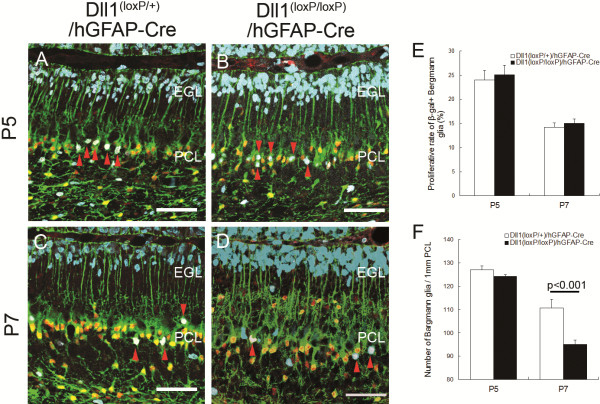
**The number of BG is reduced in Dll1**^**(loxP/loxP)**^**/hGFAP-Cre mice after P7.** (**A**-**D**) BrdU incorporation by BG in Dll1^(loxP/+)^/hGFAP-Cre (**A**, **C**) and Dll1^(loxP/loxP)^/hGFAP-Cre (**B**, **D**) mice at P5 (**A**, **B**) and P7 (**C**, **D**). The cell bodies of BG are labeled with antibodies against BLBP (green) and β-galactosidase (red) antibodies. The red arrowheads in (**A**-**D**) show proliferating BG (white). (**E**, **F**) Quantification of the number of proliferating BG (white) with incorporated BrdU (sky blue) (**E**) and BLBP-cell bodies (**F**) at P5 and P7. Scale bars: 50 μm (**A**-**D**). EGL, external granular layer; PCL, Purkinje cell layer.

Precursors of BG begin their migration at E14 and establish their layer structure into the Purkinje cell layer during the first postnatal week [1]. To address how early BG arrangement is affected in Dll1^(loxP/loxP)^/hGFAP-Cre mice, we analyzed its localization during postnatal cerebellum development, as demonstrated by in situ hybridization with *Glast* (Figure 3A-F). Surprisingly, a small number of BG is located in the external granular layer (EGL) even in the control mice at P3 (Figure 3A, G). Thus, the number of ectopic BG did not differ between the control and the mutant mice (Figure 3G). However, the number of these ectopic BG is decreased in the control mice at P5 (Figure 3H). Since Purkinje cells establish monolayer alignment during the first postnatal week, these results suggest that BG distributed in a broad cellular zone at P3 are further compacted to form a monolayer during P3 to P5. We found that ectopic BG somas in the EGL and the molecular layer (ML) were evident at P5 (Figure 3G-I). These ectopic BG, as visualized by β-galactosidase and BLBP immunostaining, were negative for BrdU labeling (Figure 4A-D), thus suggesting that they were not generated in situ but, instead, were disintegrated from the BG layer by over-migration. These results suggest that Dll1 plays a critical role in the monolayer arrangement of BG during early postnatal stages.

### Ectopic BG were eliminated by apoptosis in Dll1^(loxP/loxP)^/hGFAP-Cre mice

**Figure 5:**
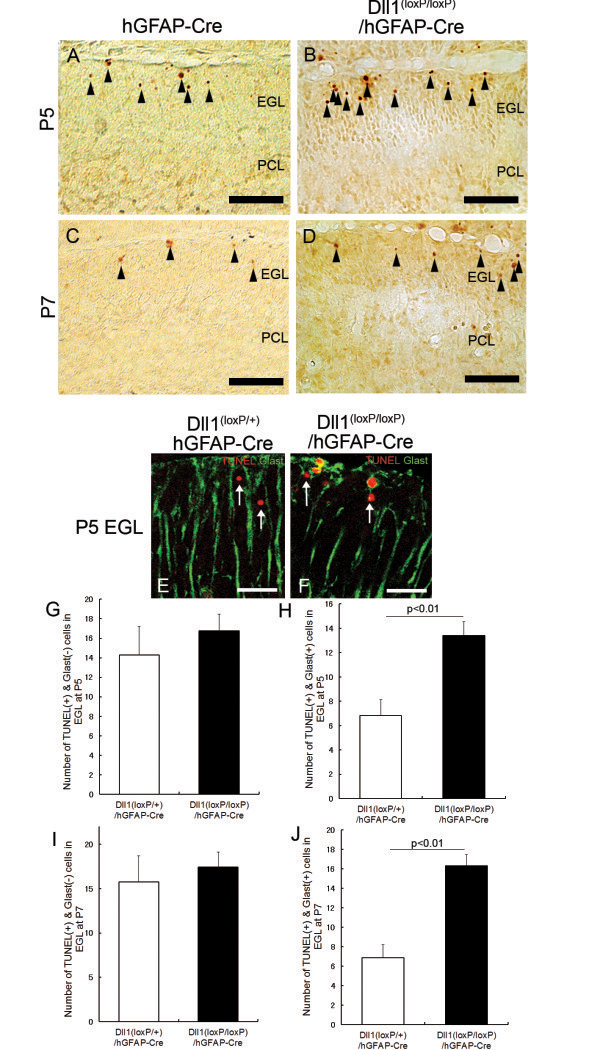
**Ectopic BG are eliminated by apoptosis in Dll1**^**(loxP/loxP)**^**/hGFAP-Cre mice.** (**A**-**D**) TUNEL staining of Dll1^(loxP/+)^/hGFAP-Cre (**A**, **C**) and Dll1^(loxP/loxP)^/hGFAP-Cre (**B**, **D**) mice at P5 (**A**, **B**) and P7 (**C, D**). The black arrowheads in (**A**-**D**) show TUNEL-positive cells. (**E**, **F**) Immunostaining for GLAST (green) and TUNEL (red) in Dll1^(loxP/+)^/hGFAP-Cre (**E**) and Dll1^(loxP/loxP)^/hGFAP-Cre (**F**) mice at P5. The white arrows indicate TUNEL-positive and GLAST-negative cells. The white arrowheads indicate TUNEL-positive and GLAST-positive cells in the EGL. (**G**-**J**) Quantification of the number of TUNEL-positive and GLAST-negative cells (**G**, **I**) and TUNEL-positive and GLAST-positive cells in the EGL (**H**, **J**) at P5 (**G**, **H**) and P7 (**I**, **J**). Scale bars: 50 μm (**A**-**D**) 20 μm (**E**-**F**). EGL, external granular layer; PCL, Purkinje cell layer.

The number of BG was reduced in adult Dll1^(loxP/loxP)^/hGFAP–Cre mice (Figure 1C). At P5, the number of BG was indistinguishable between Dll1^(loxP/loxP)^/hGFAP-Cre and control mice (Figure 4A-D, F). After P7, the number of BG was reduced in Dll1^(loxP/loxP)^/hGFAP-Cre mice (Figure 4A-D, F), this may have been caused by reduced cell proliferation or accelerated cell death. First, we evaluated the proliferative rate of BG using a BrdU labeling experiment (Figure 4A-D). The rate of BLBP^+^/β-gal^+^/BrdU^+^ (proliferative) among total BLBP^+^/β-gal^+^ BG at P5 and P7 was not significantly different in Dll1^(loxP/loxP)^/hGFAP-Cre and control mice (Figure 4A-D, E), indicating that the reduction in the number of BG in the mutants was not due to cell proliferation defects. Next, we examined the survival of mutant BG using TUNEL staining. The number of TUNEL-positive (apoptotic) cells in the mutant EGL was increased at P5 and P7 compared to control mice (Figure 5A-D). Apoptotic cells in the mutant EGL were regarded as granule cells or ectopic BG. GLAST immunostaining revealed that some of the TUNEL-positive cells in the mutant EGL were BG (Figure 5E, F). Quantitative analysis showed that only GLAST and TUNEL double-positive cells, i.e., apoptotic glial cells, were increased in the mutant mice, whereas the number of TUNEL-positive and GLAST-negative cells, i.e., apoptotic granule cells, was not significantly increased in the mutant mice at P5 and P7 (Figure 5G-J). These results suggest that the decrease in the number of BG observed in the mutant mice was due to an increase in the apoptosis of mislocalized BG.

### Ablation of Dll1 from basket and stellate cells had no effect on the maturation of BG

**Figure 6:**
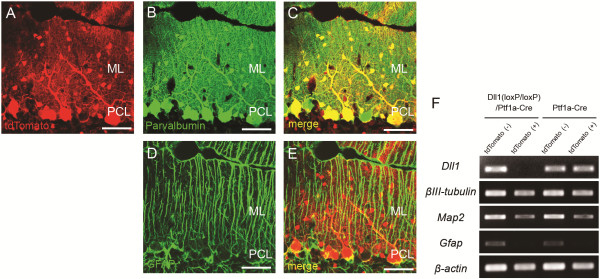
**Conditional ablation of Dll1 in cerebellar inhibitory neurons.** (**A**-**E**) Immunohistochemical images with anti-parvalbumin (**B**, **C** green) or anti-GFAP **(D**, **E** green) antibody were overlaid with tdTomato fluorescence (**A** red) in Ptf1a–Cre/ROSA–tdTomato mice. (**F**) RT-PCR analysis shows deletion of Dll1 in tdTomato-positive inhibitory neurons from Dll1^(loxP/loxP)^/Ptf1a-Cre mice. Scale bars: 50 μm (**A**-**E**). ML, molecular layer; PCL, Purkinje cell layer.

**Figure 7:**
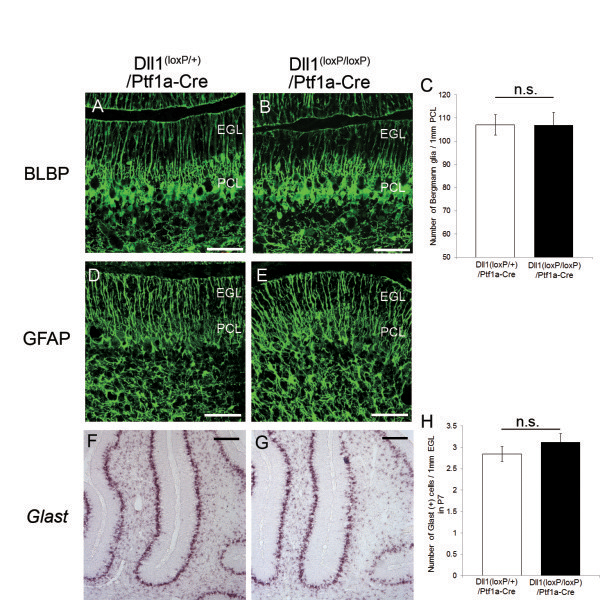
**The development of BG was normal in Dll1**^**(loxP/loxP)**^**/Ptf1a-Cre mice.** (**A**, **B**) Immunohistochemistry for BLBP (green) in Dll1^(loxP/+)^/Ptf1a-Cre (**A**) and Dll1^(loxP/loxP)^/Ptf1a-Cre (**B**) mice at P7. (**C**) Quantification of the number of BLBP-positive cell bodies. (**D**, **E**) BG labeled by anti-GFAP (green) antibody in Dll1^(loxP/+)^/Ptf1a-Cre (**D**) and Dll1^(loxP/loxP)^/Ptf1a-Cre (**E**) mice at P7. (**F**, **G**) In situ hybridization for GLAST in Dll1^(loxP/+)^/Ptf1a-Cre (**F**) and Dll1^(loxP/loxP)^/Ptf1a-Cre (**G**) mice at P7. (**H**) Quantification of the number of GLAST-positive cells in EGL at P7. n.s., no significant. Scale bars: 50 μm (**A**, **B**, **D**, **E**), 250 μm (**F**, **G**). EGL, external granular layer; PCL, Purkinje cell layer.

We previously showed that hGFAP-Cre mediated recombination occurred not only in BG but also in parvalbumin-positive interneurons (basket and stellate cells) of the molecular layer of the cerebellum [8,11]. Furthermore, Dll1 was expressed not only in BG but also in interneurons of the ML [9]. To determine if these defects in BG were mediated by a loss of Dll1 in basket and stellate cells, we generated a line of mice in which Dll1 was conditionally knocked out only in cerebellar inhibitory neurons; this was done by crossing the floxed Dll1 mice with Ptf1a-Cre mice, which in turn restricted loxP-mediated recombination in the cerebellar inhibitory neuron. The expression pattern of Cre recombinase was confirmed by crossing Ptf1a-Cre mice with ROSA-tdTomato reporter mice. In the cerebellum of Ptf1a–Cre/ROSA–tdTomato mice, tdTomato fluorescence was exclusively localized in parvalbumin-positive inhibitory neurons (Figure 6A-E). To clarify the deletion of Dll1 expression from parvalbumin-positive inhibitory neurons, we mated Dll1^(loxP/loxP)^/Ptf1a–Cre mice with ROSA-tdTomato mice and single-cell RT-PCR was then performed in the cells isolated from Dll1^(loxP/loxP)^/Ptf1a–Cre/ROSA-tdTomato or Ptf1a–Cre/ROSA-tdTomato mice. The expression of Dll1 was significantly reduced in the tdTomato-positive inhibitory neurons isolated from Dll1^(loxP/loxP)^/Ptf1a-Cre/ROSA-tdTomato mice, while Dll1 was expressed in the tdTomato-negative glial cells isolated from Dll1^(loxP/loxP)^/Ptf1a-Cre/ROSA-tdTomato mice and both tdTomato-positive inhibitory neuron and tdTomato-negative glial cell isolated from control Ptf1a–Cre/ROSA-tdTomato mice (Figure 6F). At P7 when abnormalities in BG became apparent in Dll1^(loxP/loxP)^/hGFAP-Cre mice, the number, morphology and distribution of BG were indistinguishable between Dll1^(loxP/loxP)^/Ptf1a–Cre and control mice (Figure 7). These results demonstrate that the deletion of Dll1 in the cerebellar inhibitory neurons did not replicate the phenotype of BG in the Dll1^(loxP/loxP)^/hGFAP-Cre mice, thus suggesting that Dll1 expressed in BG plays a critical role in the maturation of these cells.

### Notch-RBPj dependency of Dll1 signaling in the monolayer formation of BG

**Figure 8:**
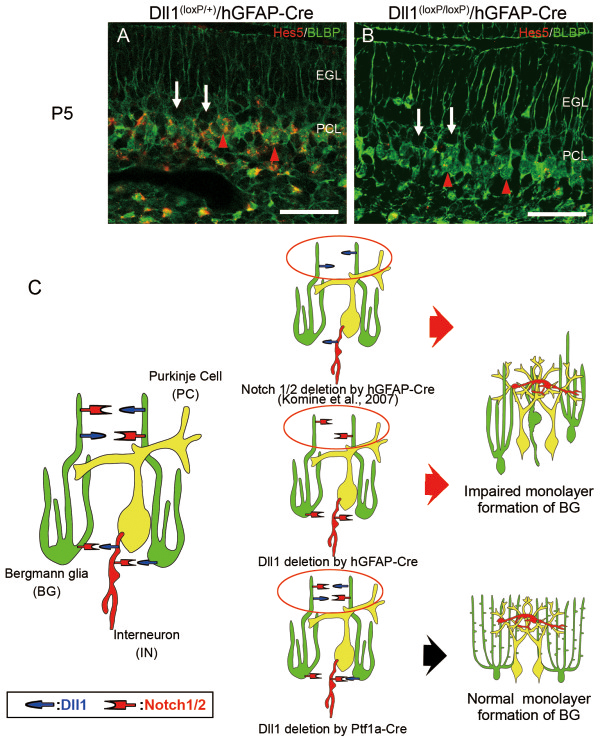
**Notch dependency of Dll1 signaling pathway in the monolayer formation and morphogenesis of BG.** (**A**, **B**) Double staining for Hes5 (red) and BLBP (green) in Dll1^(loxP/+)^/hGFAP-Cre (**A**) and Dll1^(loxP/loxP)^/hGFAP-Cre (**B**) mice at P5. White arrows show Purkinje cells. Red arrowheads show BG. (**C**) Dll1 expressed in BG (green), not in interneurons (red), plays crucial roles in the monolayer formation and morphogenesis of BG due to its Notch1/2-RBPj signaling transduction activity. Yellow cells are PCs. Scale bars: 50 μm (**A**, **B**). EGL, external granular layer; IN, interneuron; PC, Purkinje cell; PCL, Purkinje cell layer.

To examine whether the impairment of the layer formation of BG caused by loss of Dll1 is dependent on Notch-RBPj signaling, we examined the expression level of the Notch downstream target *Hes5* by in situ hybridization. *Hes5* mRNA transcripts were present in BLBP-positive cells, such as Bergmann glia and astrocytes in the white matter (Figure 8A). The expression level of *Hes5* was significantly decreased in BG of Dll1^(loxP/loxP)^/hGFAP–Cre mice (Figure 8A, B). Considering that the deletion of the Notch1/2 and RBPj in the Bergmann glia resulted in abnormal Bergmann glial positioning [8], this finding suggests that the Notch-RBPj-dependent activity of Dll1 may be responsible for the monolayer formation of BG. In conclusion, we reveal that Dll1 expressed in BG is required in the maturation of BG due to its Notch-RBPj signal transduction activity (Figure 8C).

## Discussion

In the CNS, Notch signaling is a well-known master regulator of neural stem cells and neural development [12]. In addition, Notch signaling plays instructive roles in promoting the identities of radial glia [13]. In our previous study, we revealed that Notch–RBPj signaling plays an important role in BG development by deleting Notch1 and Notch2 or RBPj. However, the ligand that activates this Notch–RBPj signaling pathway has not been identified. In this study, we found that Dll1 is the functional ligand in Notch–RBPj signaling responsible for BG monolayer formation. Since Cre recombinase is expressed in basket and stellate cells, as well as in the BG of hGFAP-Cre mice [11], abnormal layering and morphology of the BG observed in Dll1^(loxP/loxP)^/hGFAP-Cre mice could be the result from ablation of Dll1 form basket and stellate cells. To further investigate, which cell types require Dll1 for appropriate BG development, we used a Ptf1a-Cre mouse line to drive Cre-mediated deletion of Dll1 in stellate and basket cells. However, we did not observe any BG abnormalities in Dll1^(loxP/loxP)^/Ptf1a-Cre mice. Therefore, the abnormal layering and morphology of BG as observed in the Dll1^(loxP/loxP)^/hGFAP-Cre mice, was due to the deletion of Dll1 in BG, thus suggesting that Dll1-Notch1/2 interaction between adjacent BG is required for their appropriate development.

Dll1 and Jagged 1 are predominant Notch ligands expressed in the cerebellum during early postnatal development, which is the point at which Dll1^(loxP/loxP)^/hGFAP-Cre mice display abnormal BG layering [9]. The conditional ablation of Jagged 1 in the cerebellum primordium at mid-embryogenesis did not display any mislocalized BG, although ectopic granular neurons in the molecular layer were observed, as well as a reduction in the number of BG, and truncation of BG fibers [10]. Recently, the Delta/Notch-like EGF-related receptor (DNER) has been identified as a Notch ligand that is expressed on Purkinje cells. DNER-deficient mice display disorganization of BG fibers, as well as the ectopic localization of BG [14], however, abnormal BG development in DNER-deficient mice became less significant by postnatal week 3 and had improved by the adult stages, which suggests that DNER-Notch signaling is only important for the maturation of BG in the early developmental stages. Taken together, these findings suggest that Dll1-Notch signaling is required for BG monolayer formation, whereas Jagged 1-Notch or DNER-Notch signaling is not crucial for BG arrangement.

It is well known that BG start their preferential radially oriented migration at E14 towards the subpial domain, without axonal scaffold, and form their epithelium-like layer structure in the Purkinje cell layer by the first postnatal week [1]. However, the molecular mechanism underlying this event remains unknown. In this study, we demonstrated that Dll1^(loxP/loxP)^/hGFAP-Cre mice display ectopic BG after P5, whereas BG migration is normal during the embryonic stages. Furthermore, ectopic BG were negative for BrdU labeling. These results suggest that ectopic BG were not generated in situ but, instead, were disintegrated from the BG layer by overmigration. This implies that Dll1–Notch–RBPj signaling in developing BG may regulate the termination of their migration. Consistent with this finding, it has been reported that the lack of Notch and Numb causes the overmigration of peripheral glia in *Drosophila*[15]. Further studies are necessary to clarify the molecular mechanisms underlying the termination of BG.

## Conclusions

We show here that Dll1 localized to BG is required for their monolayer formation and morphological maturation in the developing cerebellum, apparently through a Notch1/2 and RBPj dependent signaling pathway.

## Methods

### Mice

hGFAP-Cre transgenic mice and Ptf1a-Cre knock-in mice, which have previously been described[16,17], were cross-bred with floxed Dll1 mice[18], to obtain conditional knockout mice. These mice were then crossed with ROSA26R mice [19] or ROSA-tdTomato mice [20] in order to examine Cre activity. All animal procedures were approved by the Animal Experiment Committee of Tokyo Medical and Dental University (0130166C).

### Astrocyte culture

Primary cerebellar astrocytes were isolated from P5 mice cerebella. Dissected cerebella were cut into small pieces and incubated in 2.5% trypsin and 0.01% DNase I, dissolved in phosphate buffered saline (PBS), for 20 min at 37°C. Trypsin activity was blocked by adding fetal bovine serum (FBS), and the tissue was triturated using a micropipette. Cells were washed with Dulbecco’s modified eagle medium (DMEM) containing 10% FBS, plated onto 10-cm tissue culture dishes, and incubated in the same culture medium.

### Immunohistochemistry and in situ hybridization

Animals were deeply anesthetized by inhalation of diethyl ether or hypothermia, and transcardially perfused with 4% paraformaldehyde (PFA) dissolved in 0.1 M phosphate-buffer (PB), the brains were then dissected out. Brains were postfixed with 4% PFA/PB overnight, and cryoprotected in 30% sucrose. Tissues were embedded in OCT compound (Sakura) and cut into 12-μm-thick sagittal sections using a cryostat. For immunohistochemistry, the sections were permeabilized with 0.5% Triton X100 dissolved in PBS, and then blocked with 5% normal horse serum and 0.3% Triton X100 dissolved in PBS. Sections were stained with either mouse monoclonal anti-β-galactosidase antibody (1:300, Promega), rabbit anti-β-galactosidase antibody (1:1000, Cappel), rabbit anti-GFAP antibody (1:1000, DAKO), mouse monoclonal anti-parvalbumin antibody (1:1000, Sigma), rabbit anti-BLBP (1:300, kindly gifted from Dr.Watanabe) antibody, rabbit anti-GLAST antibody (1:1000, kindly gifted from Dr.Watanabe), mouse monoclonal anti-NeuN antibody (1:400, Chemicon) or rabbit anti-calbindin antibody (1:1000, Sigma). Antibodies were diluted in blocking solution and incubated overnight at 4°C. The sections were then rinsed and incubated in appropriate species-specific secondary antibodies, which were tagged with Alexa-fluor 488, 568, or 633 (Molecular probes). Images were taken using confocal laser microscope (LSM510 META; Carl Zeiss).

In situ hybridization was performed on 12-μm-thick sagittal cryosections using a digoxigenin-labeled *Glast*- and *Hes5*-specific cRNA probes, as previously described [5]. Hybridized cRNA probes were detected by using alkaline phosphatase conjugated anti-digoxigenin antibody (1:1000, Roche). The sections were visualized by using NBT/BCIP solution (Roche) for *Glast* and Fast Red solution (Roche) for *Hes5*. For the double-staining of *Hes5* by in situ hybridization and BLBP by immunohistochemistry, immunohistochemistry was performed after the in situ hybridization process. Images were taken with an Olympus BX60 microscope and an Olympus DP50 digital camera or with confocal laser microscope (LSM510 META; Carl Zeiss).

### Golgi staining

Dissected brains from perfused animals were submerged in a 3% potassium dichromate solution containing 7.4% formaldehyde for 1 day in the dark, before being transferred to a 3% potassium dichromate solution and incubated for a further 2 days. Tissues were then rinsed with ultra-pure water and submerged in 2% silver nitrate solution for 4 days in the dark. After the tissues were submerged, they were embedded in agarose, and 100-μm-thick sections were cut using a Vibratome. Golgi-stained sections were viewed with an Olympus BX60 microscope, and images were acquired with an Olympus DP50 digital camera.

### BrdU staining

Postnatal mice were injected intraperitoneally with BrdU (50 μg/g body weight; Sigma) dissolved in saline. The mice were sacrificed 3 h after injection. Sagittal cryosections (12-μm-thick) were then treated with 2N hydrochloric acid at 37°C for 30 min and neutralized with 0.1 M sodium borate for 10 min. Afterwards, the sections were immunostained with an anti-BrdU antibody (Oxford Biomedical Research) and with anti-BLBP and anti-β-galactosidase antibodies.

### TUNEL staining

The Terminal deoxynucleotidyl transferase–mediated nick-end labeling (TUNEL) staining was done using the DeadEnd colorimetric TUNEL system (Promega) according to the manufacturer's instructions. Sagittal cryosections (12-μm-thick) were used for this staining technique. Horseradish peroxidase-conjugated streptavidin and diaminobenzidine were used for bright field detection, and an Alexa-fluor 568-conjugated streptavidin (Molecular Probes) and an Alexa-fluor 488-conjugated secondary antibody were used for fluorescence detection of TUNEL-positive cells, alongside immunohistochemical staining for GLAST.

### Fluorescence Activated Cell Sorting (FACS)

Cerebellar interneurons were isolated from P2 mice cerebella. Cerebella were dissected from hypothermia-induced anesthetized mice. Papain dissociation system (Worthington Biochemical Corporation) was used for the dissociation, according to manufacturer’s instructions. The cell suspension was passed through a 40 μm filter, and resuspended in Hank’s balanced salt solution. Dispersed cells were separated into tdTomato fluorescence positive and negative cells using a cell sorter (MoFlo XDP; Beckman Coulter), and each cell population was saved for RNA extraction.

### RT-PCR

Total RNA was isolated from cell culture samples or FACS sorted cells using TRIzol reagent (Invitrogen), according to the manufacturer’s instructions. Total RNA was reverse-transcribed into cDNA using SuperScript III First-Strand Synthesis System (Invitrogen), according to the manufacturer’s instructions. For cDNA detection, the following primers were used:

*Glast* FWD: AGA ATT CTG ACC TGA ACT TTG GCA GAT TA

*Glast* REV: TGG ATC CTC TTG AAA GTT GAT TTT AAA ACT

*Dll1* FWD: GAG AGA ATT CGG TCA GTG CAG TAC TGG CCT

*Dll1* REV: GAG ACT CGA GAC AGT AGC GGC CGC ACA GAC

*β-actin* FWD: ATA TCG CTG CGC TGG TCG TC

*β-actin* REV: TCA CTT ACC TGG TGC CTA GGG

*Map2* FWD: TTC CTC AGC TTG TCT CTA AC

*Map2* REV: GCT TCA GCT GTG ACT ACT TG

*βIII tubulin* FWD: ATG TCT ATG AAG GAG GTG GAC G

*βIII tubulin* REV: TCT CGG CCT CGG TGA ACT C

*Gfap* FWD: TTC TCC TTG TCT CGA ATG AC

*Gfap* REV: GGT TTC ATC TTG GAG CTT CT

### Cell counts

To examine the number of cells, at least 3 mice from each genotypic group were used and 3 sections from all individual mice were analyzed. Brain lipid binding protein (BLBP) positive cells were counted in order to assess the number of BG in the adult mice cerebella. To examine the number of BG in developing cerebellum, GLAST positive cells were counted. To examine the proliferative rate of BG, the ratio of BLBP, β-galactosidase, and BrdU triple positive cells to the total number of BLBP and β-galactosidase double positive cells in the Purkinje cell layer was calculated. In order to quantify the number of apoptotic cells in the EGL, TUNEL and GLAST double positive cells and TUNEL single positive cells were counted. The data are presented as means ± standard deviation of the mean. The P-values were calculated using Student’s *t*-test.

## Abbreviations

BG: Bergmann glia; BLBP: Brain lipid binding protein; BrdU: Bromodeoxyuridine; CNS: Central nervous system; Dll1: Delta like 1; EGL: External granular layer; GFAP: Glial fibrillary acidic protein; GLAST: glutamate aspartate transporter; IGL: Internal granular layer; Map2: Microtubule associated protein 2; ML: Molecular layer; NeuN: Neuronal nucleic protein; Ptf1a: Pancreas transcription factor 1 subunit alpha; RBPj: Recombining binding protein suppressor of hairless; PBS: Phosphate-buffered saline; PCL: Purkinje cell layer; TUNEL: Terminal deoxynucleotidyl transferase–mediated nick-end labeling.

## Competing interests

The authors declare that they have no competing interests.

## Authors’ contributions

YH, OK and KT conceived and designed the experiments. YH, OK, MN and NB performed the experiments. YH and OK analyzed the data. KH contributed reagents and materials. YH and KT wrote the paper. All authors have read and approved the manuscript for publication.

## References

[B1] Yamada K Watanabe M Cytodifferentiation of Bergmann glia and its relationship with Purkinje cells Anat Sci Int 2002 77 94 108 10.1046/j.0022-7722.2002.00021.x 12418089

[B2] Sasaki T Beppu K Tanaka KF Fukazawa Y Shigemoto R Matsui K Application of an optogenetic byway for perturbing neuronal activity via glial photostimulation Proc Natl Acad Sci USA 2012 109 20720 20725 10.1073/pnas.1213458109 23185019 PMC3528589

[B3] Sild M Ruthazer ES Radial glia: progenitor, pathway, and partner Neuroscientist 2011 17 288 302 10.1177/1073858410385870 21558559

[B4] Hatten ME Heintz N Mechanisms of neural patterning and specification in the developing cerebellum Annu Rev Neurosci 1995 18 385 408 10.1146/annurev.ne.18.030195.002125 7605067

[B5] Yamada K Fukaya M Shibata T Kurihara H Tanaka K Inoue Y Watanabe M Dynamic transformation of Bergmann glial fibers proceeds in correlation with dendritic outgrowth and synapse formation of cerebellar Purkinje cells J Comp Neurol 2000 418 106 120 10.1002/(SICI)1096-9861(20000228)418:1<106::AID-CNE8>3.0.CO;2-N 10701759

[B6] Gaiano N Fishell G The role of notch in promoting glial and neural stem cell fates Annu Rev Neurosci 2002 25 471 490 10.1146/annurev.neuro.25.030702.130823 12052917

[B7] Grandbarbe L Bouissac J Rand M Hrabé de Angelis M Artavanis-Tsakonas S Mohier E Delta-Notch signaling controls the generation of neurons/glia from neural stem cells in a stepwise process Development 2003 130 1391 1402 10.1242/dev.00374 12588854

[B8] Komine O Nagaoka M Watase K Gutmann DH Tanigaki K Honjo T Radtke F Saito T Chiba S Tanaka K The monolayer formation of Bergmann glial cells is regulated by Notch/RBP-J signaling Dev Biol 2007 311 238 250 10.1016/j.ydbio.2007.08.042 17915208

[B9] Irvin DK Nakano I Paucar A Kornblum HI Patterns of Jagged1, Jagged2, Delta-Like 1 and Delta-Like 3 expression during late embryonic and postnatal brain development suggest multiple functional roles in progenitors and defferentiated cells J Neurosci Res 2004 75 330 343 10.1002/jnr.10843 14743446

[B10] Weller M Krautler N Mantei N Suter U Taylor V Jagged1 ablation results in cerebellar granule cell migration defects and depletion of Bergmann glia Dev Neurosci 2006 28 70 80 10.1159/000090754 16508305

[B11] Komine O Nagaoka M Hiraoka Y Hoshino M Kawaguchi Y Pear WS Tanaka K RBP-J promotes the maturation of neuronal progenitors Dev Biol 2011 354 44 54 10.1016/j.ydbio.2011.03.020 21443869

[B12] Artavanis-Tsakonas S Rand MD Lake RJ Notch signaling: cell fate control and signal integration in development Science 1999 284 770 776 10.1126/science.284.5415.770 10221902

[B13] Gaiano N Nye JS Fishell G Radial glial identity is promoted by Notch1 signaling in the murine forebrain Neuron 2000 26 395 404 10.1016/S0896-6273(00)81172-1 10839358

[B14] Eiraku M Tohgo A Ono K Kaneko M Fujishima K Hirano T Kengaku M DNER acts as a neuron-specific Notch ligand during Bergmann glial development Nat Neurosci 2005 8 873 880 10.1038/nn1492 15965470

[B15] Edenfeld G Altenhein B Zierau A Cleppien D Krukkert K Technau G Klämbt C Notch and Numb are required for normal migration of peripheral glia in Drosophila Dev Biol 2007 301 27 37 10.1016/j.ydbio.2006.11.013 17157832

[B16] Bajenaru ML Zhu Y Hedrick NM Donahoe J Parada LF Gutmann DH Astrocyte-specific inactivation of the neurofibromatosis 1 gene (NF1) is insufficient for astrocytoma formation Mol Cell Biol 2002 22 5100 5113 10.1128/MCB.22.14.5100-5113.2002 12077339 PMC139771

[B17] Kawaguchi Y Cooper B Gannon M Ray M MacDonald RJ Wright CV The role of the transcriptional regulator Ptf1a in converting intestinal to pancreatic progenitors Nat Genet 2002 32 128 134 10.1038/ng959 12185368

[B18] Hozumi K Negishi N Suzuki D Abe N Sotomaru Y Tamaoki N Mailhos C Ish-Horowicz D Habu S Owen MJ Delta-like 1 is necessary for the generation of marginal zone B cells but not T cells in vivo Nat Immunol 2004 5 638 644 15146182 10.1038/ni1075

[B19] Soriano P Generalized lacZ expression with the ROSA26 Cre reporter strain Nat Genet 1999 21 70 71 10.1038/5007 9916792

[B20] Madisen L Zwingman TA Sunkin SM Oh SW Zariwala HA Gu H Ng LL Palmiter RD Hawrylycz MJ Jones AR A robust and high-throughput Cre reporting and characterization system for the whole mouse brain Nat Neurosci 2010 13 133 140 10.1038/nn.2467 20023653 PMC2840225

